# PARP inhibitor maintenance treatment for newly diagnosed ovarian cancer patients: a real-world study from China

**DOI:** 10.3389/fonc.2024.1336616

**Published:** 2024-02-02

**Authors:** Jinghong Chen, Mengpei Zhang, Kemin Li, Yuanqiong Duan, Jing Zeng, Qingli Li, Danqing Wang, Liang Song, Qintong Li, Rutie Yin

**Affiliations:** ^1^ Department of Obstetrics and Gynecology, West China Second University Hospital, Sichuan University, Chengdu, Sichuan, China; ^2^ Key Laboratory of Birth Defects and Related Diseases of Women and Children, Ministry of Education, West China Second University Hospital, Sichuan University, Chengdu, Sichuan, China

**Keywords:** ovarian cancer, poly (ADP-ribose) polymerase inhibitors, progression-free survival, adverse events, real-world study

## Abstract

**Purpose:**

This study evaluated the efficacy and safety in a real-world population of epithelial ovarian cancer (EOC) treated with poly (ADP-ribose) polymerase inhibitor (PARPi) as first-line maintenance therapy in the largest gynecologic oncology center in Western China.

**Methods:**

This study included patients newly diagnosed EOC who received PARPi as first-line maintenance therapy in West China Second University Hospital from August 1, 2018 to September 31, 2022. The primary endpoints were progression-free survival (PFS) and safety evaluated by Common Terminology Criteria for Adverse Events Version 5.0(CTCAE 5.0). The secondary endpoints were overall survival (OS) and prognostic factors influencing the PFS of patients in real world.

**Results:**

Among the eligible 164 patients, 104 patients received olaparib and 60 patients received niraparib. 100 patients (61.0%) had mutations in breast cancer susceptibility gene (BRCA). 87 patients (53.0%) received primary debulking surgery (PDS) while 77 patients (47.0%) received interval debulking surgery (IDS). 94 patients (94/164, 57.3%) achieved R0 and 39 patients (23.8%) achieved R1 after PDS/IDS. 112 (68.3%) achieved complete response (CR) after first-line chemotherapy, while 49 (29.9%) achieved partial response (PR). The median follow-up time was 17.0 months (95% CI 15.6-18.4), and the median PFS has not been reached yet. Multivariate analysis demonstrated that BRCA mutations and CR/PR after platinum-based chemotherapy were independent factors associated with prolonged PFS. Hematologic toxicity was the most common grade≥3 AE. There were no incidence of myelodysplastic syndromes/acute myelogenous leukemia (MDS/AML).

**Conclusion:**

Focusing on PARPi as first-line maintenance therapy for patients with EOC, this study represented the largest single-center real-world study in China to date. Two independent factors were identified to prolong the PFS of patients: BRCA mutated type and CR/PR after primary treatment, which should be further confirmed with long-term follow-up and large sample sizes.

## 1 Introduction

Ovarian cancer is the third most common malignancy in the female reproductive system, with mortality rate second only to cervical cancer globally. However, in developed countries, it has the highest mortality rate among female reproductive system (1). In 2020, the World Health Organization (WHO) published global cancer burden data revealing 55,342 new cases of ovarian cancer in China, accounting for 17.62% of the global incidence. In the same year, there were 37,519 deaths in China due to ovarian cancer, accounting for 18.10% of the global deaths (1, 2). Epithelial ovarian cancer (EOC) is represented by several different histology, such as serous, endometrioid, clear cell and mucinous histology, each with its own specific genetic and clinical characteristics (3). Kurman et al. provided a dualistic model for EOC according to two different carcinogenic pathways (4). Type I EOC are suggested to be relatively indolent and genetically stable tumors which consist of clearly described precursor lesions such as low-grade serous, mucinous, endometrioid, or borderline tumors at an earlier stage. In contrast, type II EOC are proposed to be high-grade, biologically aggressive tumors from their outset, where precursor lesions are not clearly described. Most patients were diagnosed with advanced EOC and there are currently no effective early detection strategies exist. The initial treatment is crucial for patients newly diagnosed with EOC as it significantly influences comprehensive management by delaying relapse, prolonging survival, and increasing the potential for cure. First-line maintenance therapy is defined as the treatment for EOC patients who have completed initial chemotherapy and achieved complete response (CR) or partial response (PR) to platinum-based survival (5). CR requires the disappearance of all target lesions and a reduction of the short diameter of all pathological lymph nodes to less than 10 mm (6). PR indicates a reduction of at least 30% in the sum of target lesion diameters compared to baseline levels.

Currently, the main maintenance therapy drugs include bevacizumab and PARP inhibitors (PARPi). Both the ICON7 study (7) and the GOG-218 study (8) found that bevacizumab could improve progression-free survival (PFS) in patients with International Federation of Obstetrics and Gynecology (FIGO) III to IV. Following the results of the SOLO1, PRIMA, PRIME and PAOLA-1 studies, the efficacy of PARPi in first-line maintenance therapy has been validated by the vast majority of EOC patients (9–13). The SOLO-1 study (10) showed that after 7 years of follow-up, 67.0% of the patients in the olaparib group were alive and half of them did not receive any subsequent treatment. The PRIMA study (11) showed that niraparib provided different degree of benefit in the first-line maintenance treatment of advanced ovarian cancer in the general population. The PRIME study (12) which performed in Asian population and used an individualized starting dose of niraparib showed a survival advantage in niraparib group regardless of surgical residual disease and biomarker status. The PAOLA-1 study (13) showed that in the HRD-positive population, OS was longer with olaparib plus bevacizumab than placebo plus bevacizumab. At 5 years, the updated PFS also showed that a higher proportion of patients with no recurrence in olaparib plus bevacizumab group. PARPi has become the standard treatment for first-line maintenance therapy in ovarian cancer. These large randomized controlled trials (RCTs) have laid a solid foundation for clinical diagnosis and treatment. However, these studies were based on strict inclusion criteria and treatment measures, which avoided bias but inevitably differed from the clinical reality (14). The real-world study (RWS) is commonly used to evaluate the efficacy of drug in real clinical practice after large RCTs, and the conclusions from RWS have better external validity (15). However, there is a lack of RWS on the PARPi in first-line maintenance therapy, especially in Chinese population. As the largest gynecological oncology center in western China, the West China Second University Hospital of Sichuan University has annually treated 450 newly diagnosed cases of EOC. Therefore, this study aimed to collect clinicopathological data from patients with EOC receiving PARPi as first-line maintenance therapy and evaluate the efficacy and safety in a real-world population from China.

## 2 Methods

### 2.1 Patients and study design

This study followed the Declaration of Helsinki and was approved by the Ethics Committee of West China Second University Hospital (approval number: 20220129). The clinicopathological data of newly diagnosed cases of EOC from August 1, 2018 to September 31, 2022 were collected. This study included: (1) age ≥ 18 years old; (2) pathologically confirmed as EOC with complete clinical and pathological data; (3) patients receiving PARPi as first-line maintenance therapy. The patients who refused follow-up or missed important clinical data were excluded. The clinicopathological data from medical records included demographics, histology, breast cancer susceptibility gene (BRCA) status, FIGO stage, neoadjuvant chemotherapy (NACT), residual diseases after primary surgery, platinum-based chemotherapy, first-line maintenance therapy details [baseline CA125 levels before PARPi, baseline computed tomography (CT) or magnetic resonance imaging (MRI) results, duration of treatment, and dose interruption/reduction/discontinuation]. Missing information will be supplemented through follow-up phone calls or face-to-face inquiries (if the patients are alive and accessible).

### 2.2 Endpoints

The primary endpoints were PFS and safety evaluated by Common Terminology Criteria for Adverse Events Version 5.0(CTCAE 5.0) (16). The secondary endpoints were OS and prognostic factors influencing the PFS of patients in real world. PFS was defined as the time from initiation of PARPi to radiographic progression according to response evaluation criteria in solid tumors (RECIST) version 1.1 (6), death from any cause, or study cutoff. OS was defined as the time from initiation of PARPi to death from any cause or study cutoff. The relevant factors included age, BRCA gene status, FIGO staging, histology, NACT, residual disease after surgery, response to chemotherapy and PARPi maintenance treatment. R0 was defined as no visible residual disease after surgery. R1 was defined as residual disease ≤1cm, and R2 was defined as residual disease >1cm. The response to chemotherapy was performed with RECIST 1.1.

### 2.3 Follow up

Follow-up was conducted through telephone interviews, outpatient visits, WeChat groups, QQ groups (an online community), and other methods to assess the survival status of patients and monitor adverse events (AEs) associated with PARPi. Safety-related data included AE terms, the highest CTCAE grade, treatment measures taken for AEs, measures taken for PARPi (dose reduction, interruption, discontinuation), and outcomes. The follow-up endpoint was disease recurrence, progression, death or the cut-off date, which was December 1, 2022.

### 2.4 Statistical analysis

For continuous variables that followed a normal distribution, they were presented as mean ± standard deviation (mean ± SD). If the variables did not follow a normal distribution, they were expressed as median (Q1, Q3). Categorical variables were presented as counts (n) and percentages (%). PFS and OS curves were described according to the Kaplan Meier method. The median follow-up time was calculated using the reverse Kaplan-Meier method. The univariate analysis associated with prolonged PFS was performed with the Log-rank test. Factors with a significance level of *P*<0.05 in the univariate analysis were included in the multivariate Cox analysis. A significance level of *P*<0.05 was used to define statistically significant differences. All statistical analyses were performed with SPSS version 25.0 software.

## 3 Results

### 3.1 Baseline characteristics

This study included a total of 164 patients, with 104 patients (104/164, 63.4%) receiving olaparib and 60 patients (60/164, 36.6%) receiving niraparib. The baseline characteristics of the patients are shown in **Table 1**. 100 patients (100/164, 61.0%) had mutations with BRCA. 77 patients (77/164, 47.0%) received NACT. 94 patients (94/164, 57.3%) achieved R0 after primary debulking surgery (PDS)/interval debulking surgery (IDS), while 17 patients (17/164, 10.4%) didn’t have residual lesions after surgery. Among these, 9 patients had no description of residual lesions in the surgical records, In 3 cases, the surgical records mentioned the presence of residual lesions but did not provide information about their size. Additionally, 5 patients underwent surgeries in other hospitals. In this study, 112 patients (112/164, 68.3%) achieved CR after first-line chemotherapy, while 49 patients (49/164, 29.9%) achieved PR. Three patients with advanced high-grade serous ovarian cancer(HGSOC) carrying BRCA mutations, who were evaluated as SD or PD after first-line chemotherapy, received olaparib treatment, and all three patients achieved R1 after PDS/IDS. A total of 40 patients (40/164, 16.1%) had an interval of more than 8 weeks between the end of chemotherapy and the start of PARPi treatment. 26 patients (26/164, 15.9%) received PARPi in combination with bevacizumab for maintenance treatment. 8 patients (8/164, 4.9%) experienced dose discontinuation due to AEs, including 7 patients in the olaparib group (3 with anemia, 1 with recurrent urinary tract infection, 1 with osteodynia, 1 with kidney failure and 1 with gastrointestinal reactions) and 1 patient in the niraparib group (grade ≥3 tachycardia).

**Table 1:** Clinicopathological characteristic in the real world.

Characteristics		Olaparib (N=104)	Niraparib (N=60)	*P*
Age (mean ± SD, years)		52.71 ± 10.33	55.42 ± 9.87	0.103
BMI (median (Q1,Q3), kg/m^2^)		22.29 (20.52-24.44)	22.95 (20.83-25.21)	0.650
Complication, n (%)	Yes	34 (32.7)	19 (32.8)	0.892
	No	70 (67.3)	41 (40.6)	
Family history, n (%)	Yes	58 (36.6)	24 (31.6)	
	No	104 (63.4)	52 (68.4)	
BRCA gene, n (%)	Wild type	9 (8.7)	52 (86.7)	**0.000**
	Mutation type	92 (88.5)	8 (13.3)	
	Unknown	3 (2.9)	0	
NACT, n (%)	Yes	53 (51.0)	24 (40.0)	0.175
	No	51 (49.00)	36 (60.0)	
The residual disease, n (%)	R0	54 (51.9)	40 (66.7)	0.277
	R1	29 (27.9)	10 (16.7)	
	R2	10 (9.6)	4 (6.7)	
	Unknown	11 (10.6)	6 (10.0)	
FIGO 2014, n (%)	I	1 (1.0)	1 (1.7)	0.929
	II	10 (9.6)	5 (8.3)	
	III	79 (76.0)	44 (73.3)	
	IV	14 (13.5)	10 (16.7)	
Histology, n (%)	HGSOC	101 (97.1)	53 (88.3)	0.057
	Endometrial	1 (1.0)	2 (3.3)	
	OCCC	0 (1.3)	2 (3.3)	
	Others	2 (1.0)	3 (5.0)	
First-line chemotherapy, n (%)	≤6 cycles	84 (80.8)	44 (73.3)	0.268
	>6 cycles	20 (19.2)	16 (26.7)	
Response to chemotherapy, n (%)	CR	69 (66.3)	43 (71.7)	0.777
	PR	32 (30.8)	17 (28.3)	
	SD	1 (1.0)	0	
	PD	2 (1.9)	0	
The interval between chemotherapy and maintenance therapy, n (%)	4-8 weeks	81 (77.9)	43 (71.6)	0.372
	>8 weeks	23 (22.1)	17 (28.4)	
Combined with bevacizumab in maintenance therapy, n (%)	Yes	22 (21.2)	4 (6.7)	**0.014**
	No	82 (78.8)	56 (93.3)	
CA125 before PARPi, n (%)	<35U/ml	101 (97.1)	54 (90.0)	0.075
	≥35U/ml	3 (2.9)	6 (10.0)	
Time of PARPi treatment, median (Q1,Q3)		15 (9-19)	9 (5-15)	**0.003**
PARPi, n (%)	Interruption	26 (25)	28 (46.7)	**0.004**
	Reduction	33 (31.7)	31 (51.7)	**0.012**
	Discontinuation	7 (6.7)	1 (1.7)	0.283

The clinicopathological data of newly diagnosed as EOC in West China Second Hospital of Sichuan University from August 1, 2018 to September 31, 2022 who took olaparib and niraparib as first-line maintenance were shown as follows. BMI, body mass index; BRCA, Breast Cancer Susceptibility Gene; NACT, neoadjuvant chemotherapy; FIGO, International Federation of Obstetrics and Gynecology; HGSOC, high-grade serous ovarian cancer; OCCC, ovarian clear cell carcinoma; CR, complete response; PR, partial response; SD, stable disease; PD, progression disease; PARPi, PARP inhibitor. The factors with a significance level of P<0.05 were bolded.

### 3.2 Efficacy

**Figure 1:**
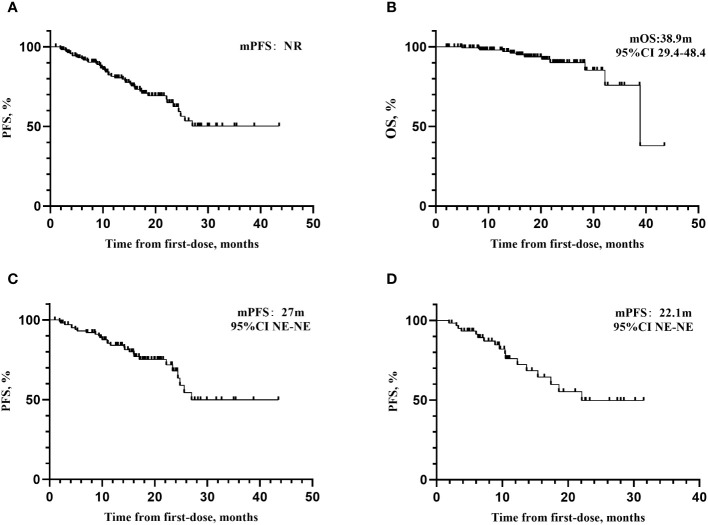
Kaplan–Meier curves for PFS and OS. **(A)** Kaplan–Meier curves for PFS. median follow-up time was 17.0 months (95%CI 15.6-18.4). As of December 1, 2022, the mPFS has not yet been reached. **(B)** Kaplan–Meier curves for OS. The mOS was 38.9 months (95%CI 29.4-48.4). **(C)** Kaplan–Meier curves for PFS of patients receiving olaparib. The mPFS for patients receiving olaparib was 27 months. **(D)** Kaplan–Meier curves for patients receiving niraparib. The mPFS for patients receiving niraparib was 22.1 months, respectively. mPFS, median progression-free survival; NE, not evaluable.

The median follow-up time was 17.0 months (95%CI 15.6-18.4). As of December 1, 2022, the patients in this study did not reach the mPFS (see **Figure 1**), and the mOS was 38.9 months (95%CI 29.4-48.4). The maturity of PFS data in this study was 26.8% (44/164), and the maturity of OS data was 7.3% (12/164). There were a case of SD and 2 cases of PD after last chemotherapy, all of whom experienced disease progression and the PFS was 10.0 months, 7.0 months, and 24.4 months, respectively.

### 3.3 Influencing factors for PFS

**Figure 2:**
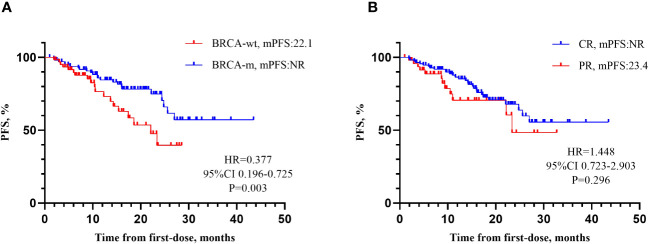
Influencing factors for PFS. **(A)** mPFS according to BRCA gene. Patients with BRCA-m demonstrated a 62.3% reduction in the risk of disease progression compared to patients with BRCA-wt (HR=0.377, 95% CI 0.196-0.725, *P*=0.003). **(B)** Response to last chemotherapy. There was no significant difference in PFS between patients who achieved CR and those who achieved PR (HR=1.448, 95% CI 0.723-2.903, *P*=0.296). It might be related to the currently immature data of PFS (26.8%).

Survival analysis was performed for PFS (see **Tables 2**, **3**). The results showed that BRCA mutations (see **Table 2**, *P*=0.030), residual diseases after PDS/IDS(*P*=0.046), the response to last chemotherapy (*P*=0.018) were associated with PFS for patients with EOC. No significant impact was found in age, family history, complications, histology, FIGO stage, cycles of chemotherapy, bevacizumab administration, the interval between chemotherapy and maintenance therapy (*P*>0.05). Above factors with a significance level of P<0.05 were included in the multivariate analysis (see **Table 3** and **Figure 2**). The results showed that the BRCA mutations and achieving CR or PR after first-line chemotherapy were independent factors influencing PFS for patients with EOC (BRCA, *P* =0.011; Response to last chemotherapy, *P*=0.043). However, there was no significant difference in PFS between patients who achieved CR and those who achieved PR (HR=1.448, 95%CI 0.723-2.903, *P*=0.296).

**Table 2:** Log-rank analysis of factors associated with prolonged PFS.

Characteristics		Log-Rank analysis		
		mPFS (95%CI)	χ^2^	*P*
Age	<65 years	NR	2.776	0.096
	≥65 years	18.6 (NE)		
Complication	Yes	25.6 (NE)	0.218	0.640
	No	NR		
Family history	Yes	NR	2.990	0.084
	No	27.0 (NE)		
BRCA gene	Wild type	22.1 (15.9-28.3)	7.014	**0.030**
	Mutation type	NR		
	unknown	17.1 (4.8-29.4)		
NACT	Yes	25.6 (21.9-29.3)	1.607	0.205
	No	NR		
The residual disease	R0	NR	6.076	**0.046**
	≥R1	24.4 (21.8-27.0)		
	unknown	NR		
FIGO 2014	I-II	NR	1.502	0.472
	III	27.0 (NE)		
	IV	24.8 (NE)		
Histology	Serous	27.0 (NE)	0.430	0.232
	Others	NR		
First-line chemotherapy	≤6 cycles	NR	1.627	0.202
	>6 cycles	24.8 (20.4-29.2)		
Response to chemotherapy	CR	NR	8.074	**0.018**
	PR	23.4 (NE)		
	SD+PD	10.0 (5.2-14.8)		
Interval between chemotherapy and maintenance therapy	4-8 weeks	NR	1.308	0.253
	>8 weeks	24.8 (NE)		
PARPi	Olaparib	27.0 (NE)	1.986	0.159
	Niraparib	22.1 (NE)		
Combined with bevacizumab in maintenance therapy	Yes	24.4 (NE)	0.082	0.774
	No	NR		
PARPi interruption	Yes	NR	3.018	0.082
	No	27.0 (NE)		
PARPi reduction	Yes	NR	3.141	0.076
	No	24.8 (NE)		

It was found that BRCA mutation、residual diseases after primary surgery, the response to last chemotherapy were associated with PFS for patients with EOC (P<0.05). mPFS, median progression-free survival; BRCA, Breast Cancer Susceptibility Gene; NACT, neoadjuvant chemotherapy; FIGO, International Federation of Obstetrics and Gynecology; HGSOC, high-grade serous ovarian cancer; OCCC, ovarian clear cell carcinoma; CR, complete response; PR, partial response; SD, stable disease; PD, progression disease; PARPi, PARP inhibitor; NE, not evaluable; NR, not reached. The factors with a significance level of P<0.05 were bolded.

**Table 3:** Multivariate analysis of factors associated with prolonged PFS.

Clinical characteristics	Multivariate analysis							
	B	SE	Wald	df	*P*	HR	95.0% CI	
							Lower	Upper
BRCA gene			9.076	2	**0.011**			
Wild type V.S. mutation type	-0.976	0.334	8.545	1	**0.003**	0.377	0.196	0.725
Wild type V.S. unknown	-0.019	0.782	0.001	1	0.981	0.981	0.212	4.542
Residual disease			5.331	2	0.070			
R0 V.S. ≥R1	0.640	0.359	3.175	1	0.075	1.897	0.938	3.837
R0 V.S. unknown	0.982	0.490	4.021	1	**0.045**	2.670	1.022	6.971
Response to chemotherapy			6.313	2	**0.043**			
CR VS PR	0.370	0.355	1.091	1	0.296	1.448	0.723	2.903
CR VS SD+PD	1.638	0.665	6.069	1	**0.014**	5.146	1.398	18.948

The BRCA gene status and achieving CR or PR after first-line chemotherapy were independent factors influencing PFS for patients with EOC. However, there was no significant difference in PFS between patients who achieved CR and those who achieved PR. BRCA, Breast Cancer Susceptibility Gene; CR, complete response; PR, partial response; SD, stable disease; PD, progression disease. The factors with a significance level of P<0.05 were bolded.

### 3.4 Safety

In this study, the safety characteristics of PARPi in real-world clinical practice were divided into olaparib group and niraparib group (see **Table 4**). In olaparib group(N=104), the most common AEs were leukopenia (67/104, 64.4%), anemia (56/104, 53.8%), loss of appetite (42/104, 40.4%) and nausea (41/104, 39.4%). The most common grade ≥3 AEs included anemia (10/104, 9.6%), thrombocytopenia (4/104, 5.3%), and leukopenia (5/104, 3.8%). In niraparib group (N=60), leukopenia (35/60, 58.3%), anemia (25/60, 41.7%) and sleeping disorders (24/60, 40.0%) were the most common AEs. Grade ≥3 AEs included thrombocytopenia (9/60, 15.0%). anemia (5/60, 8.3%), leukopenia (1/60, 1.7%), tachycardia (1/60, 1.7%) and constipation (1/60, 1.7%). There were no cases of MDS/AML.

**Table 4:** Common AEs for olaparib and niraparib in the real world.

Terms	Olaparib (N=104)		Niraparib (N=60)	
	N (%)	≥G3 (%)	N (%)	≥G3 (%)
Hematological system				
Anemia	56 (53.8)	10 (9.6)	25 (41.7)	5 (8.3)
Leukopenia	67 (64.4)	5 (3.8)	35 (58.3)	1 (1.7)
Thrombocytopenia	25 (24.0)	4 (5.3)	23 (38.3)	9 (15.0)
Gastrointestinal system				
Nausea	41 (39.4)	0	23 (38.3)	0
Vomiting	35 (33.7)	0	13 (21.7)	0
Diarrhea	8 (7.7)	0	0	0
Constipation	23 (22.1)	0	12 (20.0)	1 (1.7)
Loss of appetite	42 (40.4)	0	12 (20.0)	0
Fatigue	28 (26.9)	0	6 (10.0)	0
Infection and invasive disease				
Upper respiratory tract infection	4 (3.8)	0	1 (1.7)	0
Urinary tract infection	20 (19.2)	1 (1.0)	5 (8.3)	0
Neurological System				
Sleeping disorders	36 (34.6)	0	24 (40.0)	0
Cardiovascular System				
Tachycardia	10 (9.6)	0	11 (18.3)	1 (1.7)
Hypertension	2 (1.9)	0	5 (8.3)	0
Abdominal liver and kidney function				
Elevated transaminases	16 (15.4)	2 (1.9)	19 (31.7)	0
Elevated creatinine	19 (18.3)	0	6 (10.0)	0
Kidney failure	3 (2.9)	1 (1.0)	0	0
Others				
Muscle, skeletal and joint pain	33 (31.7)	0	12 (20.0)	0
Dermatitis, rash, photosensitivity	6 (5.8)	0	10 (16.7)	0
Oral ulcers, oral mucositis	20 (19.2)	0	5 (8.3)	0

In olaparib group, the most common AEs were leukopenia, anemia, loss of appetite and nausea. The most common grade ≥3 AEs included anemia, thrombocytopenia, and leukopenia. In niraparib group, leukopenia, anemia and sleeping disorders were the most common AEs. Grade ≥3 AEs included thrombocytopenia, anemia, leukopenia, tachycardia, and constipation. There were no cases of MDS/AML event. No additional safety signals happened.

Additionally, approximately 4.9% of patients (8/164) discontinued treatment (see **Table 5**). Among them, 2.4% (4/164) discontinued the medication due to grade ≥3 AEs (4 with anemia and 1 with tachycardia). Approximately 39.0% of patients (64/164) experienced dose reduction, with 7.9% (13/164) of them related to grade≥3 AEs, such as anemia(5/164, 3.0%) and thrombocytopenia(3/164, 1.8%). 32.9% (54/164) patients underwent dose interruption due to the most common AEs, including anemia (14/164, 8.5%), leukopenia (12/164, 7.3%) and thrombocytopenia (12/164, 7.3%).

**Table 5:** Common AEs for PARPi Interruption, Reduction, and Discontinuation.

Terms	Dose interruption		Dose reduction		Dose discontinuation	
	N (%)	≥G3 (%)	N (%)	≥G3 (%)	N (%)	≥G3 (%)
	54 (32.9)	18 (11.0)	64 (39.0)	13 (7.9)	8 (4.9)	4 (2.4)
Hematological system						
Anemia	14 (8.5)	7 (4.3)	11 (6.7)	5 (3.0)	3 (1.8)	3 (1.8)
Leukopenia	12 (7.3)	2 (1.2)	17 (10.4)	1 (0.6)	0	0
Thrombocytopenia	12 (7.3)	5 (3.0)	9 (5.5)	3 (1.8)	0	0
Bone marrow suppression	6 (3.7)	2 (1.2)	6 (3.7)	2 (1.2)	0	0
Gastrointestinal system						
Nausea	0	0	5 (3.0)	0	2 (1.2)	0
Vomiting	2 (1.2)	0	1 (0.6)	0	0	0
Diarrhea	0	0	1 (0.6)	0	0	0
Constipation	1 (0.6)	0	2 (1.2)	0	0	0
Fatigue	0	0	1 (0.6)	0	0	0
Cardiovascular System						
Tachycardia	0	0	0	0	1 (0.6)	1 (0.6)
Hypertension	1 (0.6)	0	0	0	0	0
Abdominal liver and kidney function						
Elevated transaminases	2 (1.2)	1 (0.6)	2 (1.2)	1 (0.6)	0	0
Elevated creatinine	0	0	5 (3.0)	0	1 (0.6)	0
Kidney failure	1 (0.6)	1 (0.6)	2 (1.2)	1 (0.6)	0	0
Others						
Muscle, skeletal and joint pain	1 (0.6)	0	2 (1.2)	0	1 (0.6)	0
Dermatitis, rash, photosensitivity	2 (1.2)	0	0	0	0	0
Oral ulcers, oral mucositis	0	0	0	0	0	0

Approximately 39.0% of patients had experienced dose reduction, with 7.9% of them related to Grade≥3 AEs, such as anemia and thrombocytopenia. 32.9% of patients underwent dose interruption due to the most common AEs, including anemia, leukopenia and thrombocytopenia. Approximately 4.9% of patients discontinued treatment, while 2.4% discontinued the medication due to Grade ≥3 AEs (4 with anemia and 1 with tachycardia).

## 4 Discussion

This study is a single-center real-world study with the largest sample size in China, demonstrating the effectiveness and tolerability of PARPi as first-line maintenance therapy for patients with EOC. Based on the existing data maturity, BRCA mutations and CR or PR after first-line chemotherapy were independent factors associated with prolonged PFS, which should be further confirmed with long-term follow-up and large sample sizes.

The SOLO-1 study (10), which focused on newly diagnosed advanced EOC patients with BRCA mutations and included 10 patients from our center, showed that the mPFS for patients receiving olaparib was 56.0 months after 5-year follow-up. As of the 7th year, the olaparib group did not reach the mOS. There were 32 patients in our center included in PRIME study (12). With a follow-up of 27.5 months, in the intention-to-treat (ITT) population, the mPFS was 24.8 months in niraparib group and 8.3 months in the placebo group (HR=0.45, 95% CI: 19.2-NE). Data for OS was not mature in the ITT population. By the data cutoff, 65 patients (37 in niraparib group [56.9%] and 28 in the placebo group [43.1%]) had died (HR,0.63;95% CI,0.38-1.03), and the estimated 24-month OS rate was 87.3% for niraparib and 82.7% for placebo. PRIME study identified that for patients newly diagnosed as EOC, regardless of postoperative residual diseases or biomarker status, niraparib could reduce the risk of disease progression or death compared to placebo. Based on the PRIMA study (11), after 13.8 months of follow-up, the mPFS of niraparib group was 13.8 months, showing a 38% reduced risk of recurrence or death compared to the placebo group (HR=0.62, 95% CI 0.50-0.76, *P*<0.001) in the overall population. In this real-world study, after 17.0 months of follow-up, the mPFS has not been reached, and mOS was 38.9 months (95% CI 29.4-48.4). Among them, the mPFS of olaparib group (N=104) was 27.0 months, and that of niraparib group (N=60) was 22.1 months. The mPFS of the niraparib group is comparable to that in PRIME study (12), but better than that in PRIMA study (11). It was related to that PRIMA study focused on advanced patients with high risk of recurrence, among whom 35% were FIGO IV (16.7% of patients were in this study), 66% of patients underwent NACT (40.0% of patients in this study), and 99.6% of FIGO III patients still had residual lesions after primary cytoreductive surgery (30.2% in this study). The data of OS was not mature (12/164, 7.3%). Long-term follow-up is necessary to improve the comprehensiveness and reliability of survival data.

Genetic testing is considered crucial in the assessment of familial genetic risk. The first edition of the National Comprehensive Cancer Network (NCCN) Ovarian Cancer Guidelines in 2023 re-emphasized the significance of BRCA gene testing for all non-mucinous ovarian cancer patients upon their first pathologically diagnosis. It was also highlighted the necessity of HRD testing for BRCA wild type (BRCA-wt) patients (17). However, it was found that in the first-line maintenance treatment, the olaparib group had 88.5% of BRCA mutated type (BRCA-m) patients, while the niraparib group had 86.7% of BRCA-wt patients. The difference in BRCA gene status between the two groups was statistically significant (*P*<0.001). The reason for this difference is that the first edition of the NCCN guidelines in 2019 (18) recommended the use of olaparib for BRCA-m patients. However, the first edition of the NCCN guidelines in 2020 (19) recommended niraparib for all newly diagnosed advanced EOC patients. It reflected a strict adherence to guidelines and the emphasis on patient education in our center. In this study, patients with BRCA-m demonstrated a 62.3% reduction in the risk of disease progression compared to patients with BRCA-wt (HR=0.377, 95% CI 0.196-0.725). In the PRIME study (12), mPFS with niraparib was not reached in patients with germline BRCA-m and 19.3 months in patients without germline BRCA-m, respectively; For patients receiving niraparib, the mPFS was not reached with homologous recombination deficient (HRD) and 16.6 months with homologous recombination proficient, respectively. In the PRIMA study (11), the mPFS for patients with BRCA-m, BRCA-wt/HRD-positive and BRCA-wt/HRD-negative were 22.1 months, 19.6 months, and 8.1 months, respectively. It demonstrated that HRD-negative patients derived significantly less benefit compared to those with BRCA-m and HRD-positive patients. Additionally, only 23% of BRCA-wt patients in this research underwent HRD testing, which could be attributed to several factors. Firstly, our center was located in a less economically developed region in the western China. The cost of HRD testing was expensive, making it unaffordable for many patients. Moreover, there were no approved HRD testing kits available for clinical use in China and some HRD tests had false-positive and false-negative results (9). Therefore, it is essential to promote greater access to HRD testing kits to support clinical practice and research.

Additionally, except for BRCA/HRD testing, technologies of proteomics play a gradually important role in ovarian cancer. Proteomics analysis of ovarian cancer, as well as their adaptive responses to therapy, can uncover new therapeutic choices, which can reduce drug resistance and potentially improve patient outcomes (20). Paulovich, et al. performed a proteogenomic analysis of untreated HGSOCs (chemotherapy-sensitive and refractory) which identified a highly specific 64-protein signature to predict a subpopulation of refractory HGSOCs (21). In addition, they also identified 5 different HGSOC subtypes based on protein expression in the pathway, which may represent different resistance mechanisms and serve as potential therapeutic targets. Consequently, we do believe that proteomic analysis will be a dawn of a new era for the discovery of new biomarkers for diagnosis and prognosis of EOC patients.

In this study, the mPFS for patients with CR, PR, SD+PD after chemotherapy were not reached, 23.4 months, 10.0 months, respectively. CR or PR after first-line chemotherapy was an independent factor associated with prolonged PFS. However, there was no significant difference in PFS between patients who achieved CR and those who achieved PR (HR=1.448, 95% CI 0.723-2.903, *P*=0.296). It might due to the currently immature data of PFS (26.8%). In a study with 84 ovarian cancer patients in the real-world setting, there was no significant difference in PFS between patients with CR and patients with PR (HR=0.520, 95% CI 0.115-2.339, *P*=0.394) (22). Another study including 76 EOC patients found that CR after first-line chemotherapy was an independent factor influencing PFS. The PR group had a higher risk of disease progression compared to the CR group (HR=3.208, 95% CI 1.278-8.056, *P*=0.013) (23). Additionally, during the data collection process, we identified 3 BRCA-m patients with HGSOC who were assessed as SD (n=1) and PD (n=2) after first-line chemotherapy and subsequently received olaparib treatment. All three patients had R1 after PDS/IDS. At the end of the follow-up period, they all experienced disease progression, with PFS of 10.0 months, 7.0 months, and 24.4 months, respectively. It highlighted the clinical challenge of using PARPi for patients who did not achieve CR or PR after first-line platinum-based chemotherapy but had high-risk factors. For patients who do not meet the recommended scope of clinical guidelines, it is crucial to make clinical decisions based on a comprehensive evaluation of the individual clinical situation and patient-centered care.

NACT could increase the probability of satisfactory cytoreductive surgery, reduce perioperative complications, and improve the quality of life for EOC patients. However, compared to PDS, NACT followed by IDS did not significantly improve the OS of patients (24–26). In this study, 47.0% of the patients received NACT. The mPFS in the NACT+IDS group was 25.6 months, while that in the PDS group was not reached. There was no statistically significant difference in PFS between the two groups (*P*>0.05). The PRIMA study (11) showed that in patients with NACT+IDS, the mPFS of niraparib group was 13.9 months, and the risk of disease progression or death was reduced by 41% compared with the placebo group (HR=0.59, 95%CI 0.41-0.76). The mPFS in NACT group in this study was longer than that in the PRIMA study. This could be attributed to the fact that the PRIMA study enrolled patients with high risk of recurrence. Additionally, a *post hoc* analysis of the PRIMA study revealed that patients with PDS showed a mPFS of 13.7 months in the niraparib group (N=158) compared to 8.2 months in the placebo group (N=78, HR=0.67, 95%CI 0.47-0.96). In the NACT+IDS group, the mPFS of the niraparib group (N=316) were 6 months longer than that of the placebo group (N=165, 14.2 months V.S. 8.2 months, HR=0.57, 95% CI 0.44-0.73) (27). Indeed, regardless of whether NACT was administered or not, niraparib showed the ability to improve the PFS of patients. However, there was limited research on whether NACT could enhance the effectiveness of PARPi as first-line maintenance therapy or not. It is crucial to further explore the impact of NACT on the prognosis of EOC patients with larger sample sizes.

In the overall management of advanced ovarian cancer, no macroscopic residual lesions after surgical treatment (R0) is important to improve the prognosis of patients and avoid the occurrence of platinum resistance (28). In the multivariable analysis, there was no significant difference in PFS among groups with different macroscopic residual lesions (*P*=0.07). It might due to the data immaturity of PFS (26.8%) and small sample size. Additionally, 9 cases lacked descriptions of residual disease in the surgical records, 3 cases only mentioned the presence of residual disease without specifying the size, and 5 cases underwent surgeries in other hospital. Clinical physicians should be reminded to provide detailed and explicit records of the presence of residual disease, its location, size, and other relevant information after surgery. Furthermore, CR after chemotherapy may potentially weaken the impact of R0 resection on patient prognosis. Therefore, long-term follow-up is needed to confirm the results of *post hoc* analysis. Nevertheless, R0 resection remains a cornerstone in the comprehensive management of advanced ovarian cancer, which is a crucial factor in prolonging the time to disease recurrence, avoiding resistance, and improving prognosis of patients.

In the PAOLA1 study (13), after a median follow-up time of 22.9 months, the mPFS of olaparib combined with bevacizumab in the general population was 22.1 months, and the risk of disease progression or death was reduced by 41% compared with the placebo plus bevacizumab group (HR=0.59, 95% CI 0.49-0.72, *P*<0.001). The 2022 ESMO meeting updated the 5-year PFS rate of olaparib combined with bevacizumab in HRD-positive patients. The risk of disease progression or death was reduced by 59% compared with placebo combined with bevacizumab (46.1% V.S. 19.2%, HR= 0.41, 95%CI 0.32-0.54), and the 5-year OS rate of HRD-positive patients was 65.5% (HR=0.62, 95%CI 0.45-0.85) (29). The OVARIO study (30) presented its latest data at the 2022 Society of Gynecologic Oncology (SGO) conference. With a median follow-up time of 28.7 months, the combination of niraparib with bevacizumab demonstrated a mPFS of 19.6 months (95% CI 16.5-25.1) in the overall population. In this study, the patients who received a combination of PARPi and bevacizumab were specifically those with residual disease ≥R1 after surgery or those with other high-risk factors for recurrence in ovarian cancer. None of the 24 patients reached the mPFS or mOS, which suggested a potential beneficial trend of PARPi in combination with bevacizumab for patients with residual disease ≥R1 or those with high risks of recurrence. Additional RWS with larger sample sizes and longer follow-up periods are necessary in the clinical practice.

No new AEs were found in this study. Hematologic toxicity was the most common grade≥3AE, which was the main cause of dose reduction, interruption and discontinuation. It may be related to the physiological functions of PARP enzyme, except for DNA repair. For example, PARP1 regulates cell differentiation in the bone marrow or hematopoietic system (31), while PARP2 plays a role in regulating erythropoiesis (32). Additionally, PARP1 is expressed in the megakaryocyte lineage to regulate the formation of platelets (33). The incidence of grade≥3 anemia was 9.6% in olaparib group and 8.3% in niraparib group, compared to 22.0% in SOLO1 study (34), 31.6% in PRIMA study (11), and 18.0% in PRIME study (12). The incidence of severe anemia in our center was relatively low, which could be attributed to the individualized starting dose administration, and the rigorous monitoring and management of complete blood counts. The incidence of grade ≥3 thrombocytopenia in our study was closely consistent with the data from the PRIME study (15.0%V.S. 14.1%) (12), both of which were based on the Chinese population.

There was no case of MDS/AML in this study. However, the incidence of myeloid neoplasms in SOLO1 study after 7-year follow-up was 1.5% while that in PRIMA study after 3.5-year follow-up was 1.2% and in PAOLA-1 study after 5-year follow-up was 1.7% (10, 11, 13, 35). As a delayed AE, the median latency period of the occurrence of MDS/AML after taking PARPi was 17.8 months, which was considered as a critical window period for the development of myeloid neoplasms after PARPi (36). Additionally, persistent cytopenia is considered as an early warning sign. Active surveillance, differential diagnosis, and prompt hematological referral are crucial for MDS/AML (35).

## 5 Limitation

1) The sample size for first-line PARPi maintenance therapy was not large enough as expected. It might be related to the fact that the center was in a less economically developed region in the western China, PARPi and BRCA testing were both expensive in the patient’s cognition when PARPi were first recommended by NCCN guideline in 2019. With the indications of drugs added to medical insurance, the acceptance of PARPi has been gradually increased.
2) It was difficult to establish a control group. As a single-arm retrospective RWS, the efficacy of PARPi could only be compared with external controls, such as SOLO-1, PRIMA, PRIME studies.
3) Some patients just had a follow up of 3 months. Hence, we provided the data maturity as a reference. However, insufficient data maturity may result in less significant statistical results and inaccurate estimates of the power. Long-term follow-up is necessary to accumulate more survival-related data and further analyze the factors that influence the treatment efficacy in patients.
4) Due to the high cost of HRD testing and the lack of availability of relevant domestic HRD testing kits, some patients did not complete HRD testing. Therefore, this study did not conduct an analysis about HRD-related data.
5) The collection of safety data had some limitations, such as the investigator’s assessment of the causal relationship between AEs and PARPi, and the treatment measures taken for the AEs. Further standardization is needed in the collection and administration of safety data.

## 6 Conclusion

This study represents the largest single-center real-world study conducted in China to date, focusing on the use of PARPi as first-line maintenance therapy for patients with EOC. The BRCA mutation status and the achievement of CR/PR in first-line chemotherapy were identified as independent factors influencing the PFS of patients. There have been no cases of MDS/AML by the study cuf-off.

## Data availability statement

The datasets presented in this article are not readily available because the data generated in this study are not publicly available, which may compromise patient privacy or consent. Requests to access the datasets should be directed to RY, yinrutie@scu.edu.cn.

## Ethics statement

The studies involving humans were approved by Medical Ethics Committee of West China Second University Hospital, Sichuan University. Ethical Lot Number 20220129. The studies were conducted in accordance with the local legislation and institutional requirements. The human samples used in this study were acquired from Electronic medical record system. Written informed consent for participation was not required from the participants or the participants’ legal guardians/next of kin in accordance with the national legislation and institutional requirements.

## Author contributions

JC: Data curation, Formal analysis, Investigation, Methodology, Writing – original draft. MZ: Data curation, Writing – original draft. KL: Formal analysis, Writing – original draft. YD: Investigation, Writing – original draft. JZ: Methodology, Writing – original draft. QL: Project administration, Resources, Writing – original draft. DW: Project administration, Resources, Writing – original draft. LS: Resources, Writing – original draft. QL: Conceptualization, Supervision, Writing – review & editing. RY: Conceptualization, Funding acquisition, Project administration, Resources, Supervision, Validation, Writing – review & editing.
